# Phylogeography of *Daphnia magna* Straus (Crustacea: Cladocera) in Northern Eurasia: Evidence for a deep longitudinal split between mitochondrial lineages

**DOI:** 10.1371/journal.pone.0194045

**Published:** 2018-03-15

**Authors:** Eugeniya I. Bekker, Dmitry P. Karabanov, Yan R. Galimov, Christoph R. Haag, Tatiana V. Neretina, Alexey A. Kotov

**Affiliations:** 1 Laboratory of Aquatic Ecology and Invasions, A. N. Severtsov Institute of Ecology and Evolution of Russian Academy of Sciences, Moscow, Russia; 2 Laboratory of Fish Ecology, I. D. Papanin Institute for Biology of Inland Waters of Russian Academy of Sciences, Borok, Yaroslavl Area, Russia; 3 Laboratory of Experimental Embryology, Koltzov Institute of Developmental Biology of Russian Academy of Sciences, Moscow, Russia; 4 CEFE, CNRS, Univ Montpellier, Univ Paul Valéry Montpellier 3, EPHE, IRD, Montpellier, France; 5 N.A.Pertsov White Sea Biological Station, Biological Faculty, M.V. Lomonosov Moscow State University, Moscow, Russia; 6 Kazan Federal University, Kazan, Russia; National Cheng Kung University, TAIWAN

## Abstract

Species with a large geographic distributions present a challenge for phylogeographic studies due to logistic difficulties of obtaining adequate sampling. For instance, in most species with a Holarctic distribution, the majority of studies has concentrated on the European or North American part of the distribution, with the Eastern Palearctic region being notably understudied. Here, we study the phylogeography of the freshwater cladoceran *Daphnia magna* Straus, 1820 (Crustacea: Cladocera), based on partial mitochondrial *COI* sequences and using specimens from populations spread longitudinally from westernmost Europe to easternmost Asia, with many samples from previously strongly understudied regions in Siberia and Eastern Asia. The results confirm the previously suspected deep split between Eastern and Western mitochondrial haplotype super-clades. We find a narrow contact zone between these two super-clades in the eastern part of Western Siberia, with proven co-occurrence in a single lake in the Novosibirsk region. However, at present there is no evidence suggesting that the two mitochondrial super-clades represent cryptic species. Rather, they may be explained by secondary contact after expansion from different refugia. Interestingly, Central Siberia has previously been found to be an important contact zone also in other cladoceran species, and may thus be a crucial area for understanding the Eurasian phylogeography of freshwater invertebrates. Together, our study provides an unprecedented complete, while still not global, picture of the phylogeography of this important model species.

## Introduction

Planktonic water fleas (Crustacea: Cladocera) are attractive models for phylogeographic investigations [1–4]. Indeed, planktonic samples usually contain many (tens, hundreds or even thousands) of specimen of several cladoceran taxa. Moreover, freshwater animals are highly suitable for phylogeographic studies because they have well-defined populations in water bodies separated by unsuitable habitat [5].

Many cladocerans are thought to be distributed across the entire Holarctic region, with populations being common throughout the range, which would potentially make them excellent models for Pan-Holarctic phylogeographic studies. However, our conclusions on their large distribution ranges may be too preliminary, because each taxon may in fact consist of several “cryptic” species with more local distribution ranges [6–12]. One major difficulty for phylogeographic studies of potentially Pan-Holarctic species is the need to cover large territories by adequate sampling. For instance, clustered sampling of species with uniform isolation by distance may lead to erroneous identification of genetic clusters, which may falsely be interpreted as cryptic species [13].

Most previous phylogeographic studies of cladocerans concentrated on North America [1] and Europe [14–16]. More recently, several studies on the Asian regions were published [17–20]. However, North-Eastern Eurasia is still strongly underrepresented, even in studies that otherwise cover a large geographic region [21–28]. This situation is likely explained by the logistic problems with access to different parts of Siberia, a huge territory with almost no human infrastructure. During the last decade, our team has conducted a special sampling program in this region (e.g., [29,30]). The genetic analysis of these samples has revealed phylogeographic patterns specific to Siberia and the northern half of Asia [31–33]. For two taxa (*Moina* and *Chydorus*), we demonstrated the existence of two faunistic super-complexes: (1) a European-Western Siberian super-complex and (2) a Eastern Siberian-Far Eastern super-complex, with a transition zone in the Yenisey basin, approximately near the boundary between Western and Eastern Siberia [31]. We also identified a special role of a Beringian region (including the Chukot Area of Russia, Kamchatka Peninsula, Alaska and surrounding territories) as a center of dispersion of cladocerans in Eurasia [32]. The generality of these patterns needs further verification, based on analyses of other taxa, as the identification of general patterns has the potential to strongly improve our understanding of the history of the whole freshwater fauna of Northern Eurasia.

Among numerous taxa of the cladocerans, one particular species, *Daphnia* (*Ctenodaphnia*) *magna* Straus, 1820, is the object of hundreds of scientific publications per year. Besides being used for evolutionary, ecological, and physiological studies [34,35], it is also a popular model for toxicological studies [36,37], and is used in aquaculture, mainly as food for aquarium fish and for juveniles of commercial fish species. Earlier studies on the phylogeography of *D*. *magna* have concentrated on Europe [14,38,39] and Turkey [20]. But *D*. *magna* is a common species also in the Middle East, Central Asia, several regions of Western and Eastern Siberia, China, and Japan (though the latter could be a dubious record, D. Ebert, personal communication), and is also found in North Africa, South Africa, and North America [40,41,9]. Several strongly divergent *COI* sequences have been reported from some of these regions [42,43], though it is unknown whether the strong divergence is explained by the existence of two or more highly divergent groups within the taxon or due to limited sampling of localities with intermediate haplotypes.

The aim of our study is to address these taxonomic, biogeographic, and phylogenetic knowledge gaps for *Daphnia magna* in the Palaearctic, with special reference to the Eastern Palearctic region. Through concentrating on this region we also aimed at increasing our knowledge of potentially general phylogeographic patterns among cladocerans (and perhaps other freshwater-inhabiting taxa) in the Palaearctic. We use partial COI sequences of a large number of specimens from many populations spread across the Northern part of the region, with fewer specimens also from other parts of the region (North Africa, Middle East, Caucasus) and from North America. These samples are complemented with already published data on specimens of known origin.

## Material and methods

### Field collection

We collected a total of 174 specimens from 67 populations of *Daphnia magna*, three specimens from a single population of *D*. *similis*, and 17 specimens from 14 populations of *D*. *sinensis* (S1 Table). The latter two species also belong to the subgenus *Ctenodaphnia* and are used as outgroup species. Specimens were collected with plankton nets (diameter: 20–40 cm, mesh size: 30–50 μm) or rectangular dip nets (handle length: 0.5–2 m, width: 0.2–0.3 m, mesh size: 30–50 μm) in different types of freshwater habitats and preserved in 90–96% ethanol. Before DNA extraction, each specimen was preliminarily identified to species level (*D*. *magna* or other species of the subgenus *Ctenodaphnia*), based on its morphology according to existing identification keys [9,33,41].

### DNA sequencing

Genomic DNA was extracted using the Wizard Genomic DNA Purification Kit (Promega Corporation, Madison, WI, USA) according to the manufacturer’s instructions, and fragments were amplified with primers listed in Table 1. Note that, in addition to COI, we amplified fragments of an additional mitochondrial and three nuclear genes (Table 1) in a subset of samples. However, the results on these additional fragments were inconclusive due to insufficient resolution and sample size. We therefore present them only in S1 Text.

**Table 1:** Primers used to amplify mitochondrial and nuclear fragments used in this study.

Gene fragment	Primers
5’ region of the mitochondrial cytochrome c oxidase subunit I (*COI*)gene	jgLCO1490+jgHCO1490 [44], Dm_FL+RL [45], ZooplF+R [42], *COI*-F+R [43]
5’ region of the mitochondrial rDNA *16S* gene	*16S*in-F + *16S*in-R [17]
5’ region of the nuclear rDNA 18S gene	18a1 +700R [46]
5’ region of the nuclear heat shock protein 90 kDa gene	HSP-90_F+R [47]
Region of the nuclear histone H-3 gene	*H3*F+R [48]

Polymerase chain reactions (PCR) were carried out in a total volume of 25 μl, consisting of 2 μl of genomic DNA, 8.5 μl of bi-distilled water, 1 μl of each primer (10 mM) and 5 μl PCR 5x Taq ScreenMix-HS (Evrogen, Moscow, Russia). The PCR conditions for the amplification followed those described in the papers mentioned in Table 1. The PCR products were electrophoresed together with a 0.1–3 kb DNA ladder (SibEnzyme, Novosibirsk, Russia) on a 1.5% agarose gel stained with ethidium bromide and visualized under UV light. The obtained PCR products were reprecipitated at room temperature with addition of ethanol (final concentration 70%) and ammonium acetate (final concentration 125 mM). The DNA precipitate was washed with 70% ethanol, dried, and dissolved in bi-distilled water. About 0.3 pmol of the PCR product and 3.2 pmol of the relevant primer were used for Sanger sequencing. Each PCR product was sequenced bi-directionally on an ABI 3730 DNA Analyzer with the ABI PRISM BigDye Terminator v. 3.1 sequencing kit (Applied Biosystems, USA). A single consensus sequence was assembled based on the forward and reverse sequences using CodonCode Aligner v. 6.0.2 (CodonCode Corp, USA). DNA sequences were submitted to the NCBI GenBank database (S1 Table).

### Phylogenetic analyses

The authenticity of the sequences was verified by BLAST comparisons with published *D*. *magna* sequences [49]. *COI* Sequence JF821194 from Turkey was excluded from the analysis because a preliminary tree suggested that this sequence belongs to a separate clade, which, however, could be an artifact and needs further confirmation. Sequences were edited and assembled in uGene v.1.26 [50]. The original sequences of the present study (S1 Table) and sequences from previous publications [51,52,53,54,55,56,57,58,59,60] deposited to the GenBank (S2 Table) were used for multi-sequence alignments. The sequences were first automatically aligned using the T-Coffee algorithm [61] with default options of the uGene package. Due to the strong variability of the *COI* flanking regions in Crustacea, no universal primers for barcoding exist, in contrast, for example, to fishes [62]. As a consequence, sequences from GenBank had been amplified with varying primers, explaining the variation in fragment lengths. Hence, all *COI* sequences were cropped to the minimal overlapping length of 563 bp.

To analyze the genetic variation among samples, we estimated the following parameters for each gene fragment and for each species separately: number of haplotypes (Nh), number of variable (polymorphic) sites (Nv), number of parsimony informative sites (Np), haplotype diversity (Hd), nucleotide diversity (Pi), and average number of nucleotide differences (k) [63,64]. We also carried out neutrality tests and assessed mismatch distributions. All analyses were performed in DnaSP v.5.1 [65] and MEGA v.7 [66].

The best-fitting model of nucleotide substitution was selected using the ModelFinder web application [67], based on likelihood scores for 154 different models and the Bayesian information criterion [68]. Within- and among-clade distances were calculated in MEGA using *p*-distance [69]. Phylogenetic trees were constructed using *D*. *similis* and *D*. *sinensis* as outgroup species. A maximum likelihood (ML) phylogenetic reconstruction was performed using the IQ-TREE web server [70] and ultrafast bootstrap [71] resampled 10000 times. Maximum parsimony (MP) analyses were performed in PAUP*4.0a152 [72]. A heuristic MP searches was done using equal weighting, 10 random sequence addition replicates and TBR branch swapping. Non-parametric bootstrapping was performed to assess the nodal support, using 1000 pseudoreplicates for MP. Bayesian analyses (BI) were performed in BEAST v2.4.6 [73]. Six independent Markov chain Monte Carlo (MCMC) analyses were run simultaneously for 50 million generations and sampled every 1000 generations. The first 50% of the generations were discarded as burn-in. A 50% majority rule consensus tree was generated from the remaining trees, and the posterior probability of each node was estimated as the percentage of trees recovering any particular node. MP and BI analyses were performed on the computer cluster CIPRES Science Gateway v.3.3 [74].

To establish whether there was any evidence for cryptic species, we performed a Bayesian generalized mixed Yule coalescent (bGMYC) analysis [75] with a threshold of 0.5, using the last 100 trees of the BEAST MCMC file and the GMYC-web server [76]. Furthermore, we used our haplotypes to construct a network in the program PopART [77] to show relationships among the individuals sampled from different locations. The TCS algorithm was selected for this network, based on the implemented statistical parsimony [78]. Second, the Network package (version 5, http://www.fluxus-technology.com) was used to construct a median-joining haplotype network with Steiner maximum parsimony post-processing [79], putting equal weight on each variable nucleotide site. Before constructing this network, data were processed using the star contraction algorithm [80] to reduce of number of similar haplotypes and hence to reduce the complexity of the network. As an additional test for presence of distinct clades, we used the program ABGD [81] to assess *p*-distances [69] with 100 replicate steps in this reduced-complexity network.

The reduced-complexity network was also used for a nested clade analysis, which identifies significant phylogroups, and to test for historical demographic processes within the major phylogroups. The nested clade phylogeographic analysis (NCPA) was performed in the program ANeCA v.1.2 [82] with integrated module GeoDis v.2.6 for calculation based on a new approach to the interpretation of biological processes according to [83]. Subsequently, to test for neutrality within individual clades, Tajima’s *D* tests [84] and Fu’s *FS* tests [85] were performed in Arlequin v.3.5 [86] with 1000 permutations. We also performed R2-tests [87] in DnaSP, again with 1000 permutations. Mismatch distributions were investigated for some groups to evaluate a model of exponential population growth [88]. For the latter, a goodness of fit test was performed, using a parametric bootstrap approach based on the sum of squared deviations (SSD) between the observed and simulated mismatch distributions [89]. The demographic parameter Tau was estimated using a generalized nonlinear least square approach, and the confidence interval of this parameter was computed using parametric bootstrap with 1000 replicates in Arlequin.

Divergence times were estimated using a relaxed molecular clock approach with uncorrelated lognormal distributions of branch rates in the program BEST v.1.6.1, following methods and calibrations of [90]. A second variant of this analysis was done using the additional calibration point of [91] for the *Dapnia*/*Ctenodaphnia* split (145 MYA), which suggests that the speed of nucleotide substitutions is significantly lower than suggested by [90].

## Results

### Genetic diversity

**Fig 1:**
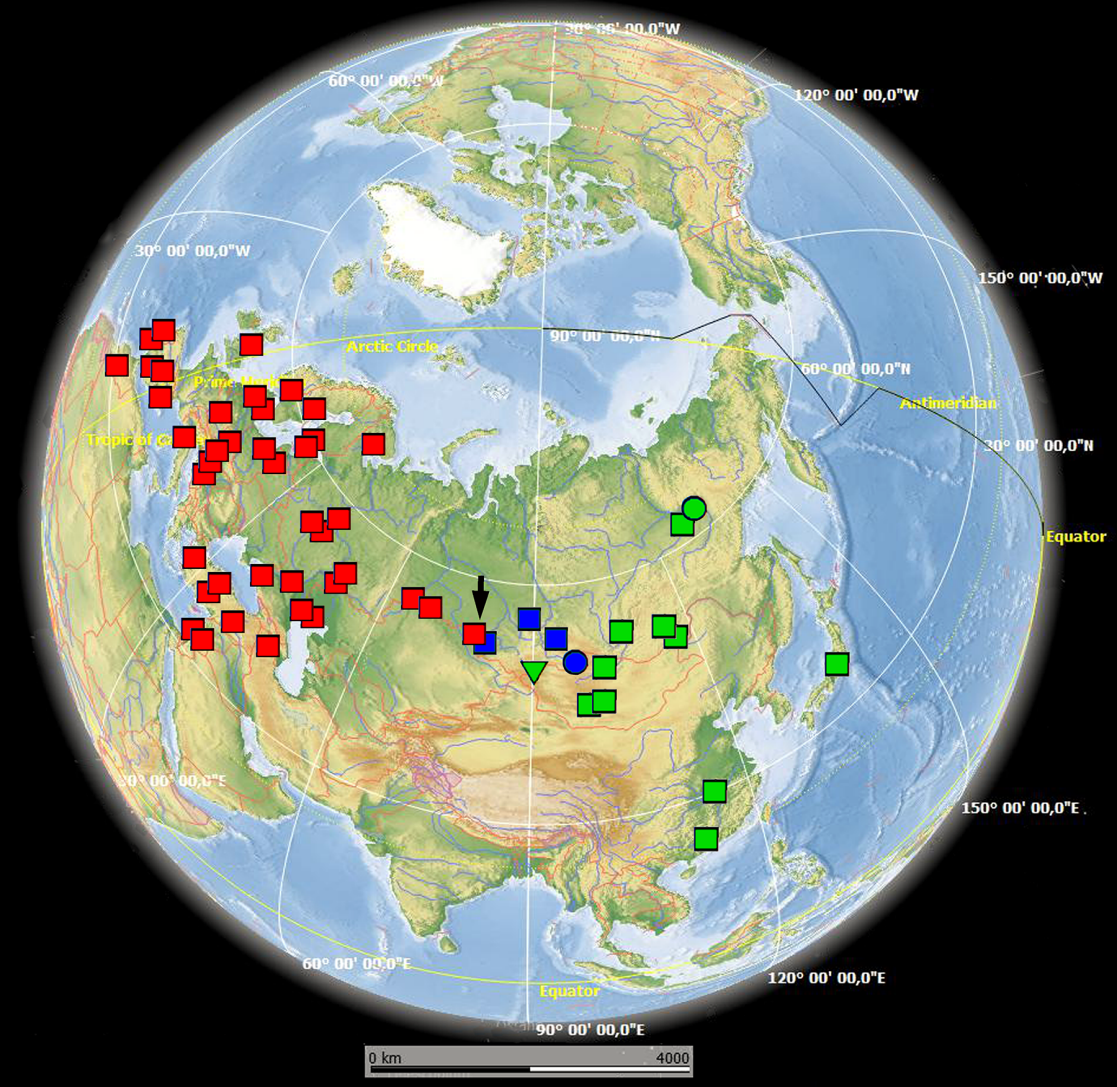
Sampling sites and distribution of major *COI* haplotype clades for Eurasian accessions of *Daphnia magna* (both original and sequences retrieved from GenBank). Colors and symbols correspond to those used in subsequent figures. The base map was obtained from the open domain plain map available at https://marble.kde.org/.

We obtained 174 original *COI* sequences of three *Daphnia* species (*D*. *magna*, *D*. *similis*, *D*. *sinensis*) from different regions of the Palaearctic (S1 Table, Fig 1). They were analyzed together with 609 *COI* sequences obtained from Genbank (S2 Table). As we found that these haplotypes belong to two strongly divergent clades (“super- clades” A and B, see below), we analyzed genetic diversity in the whole sample as well as within each of the two super-clades (S3 Table) and carried out neutrality tests and assessed mismatch distributions also for each super-clade separately (S4 Table).

The genetic diversity at *COI* was high in the entire sample as well as in super-clades A and B. Haplotypic diversity was higher in super-clade A (mainly European populations) than in super-clade B (mainly Asian populations), possibly explained by the wider geographic distribution of super-clade A. However, nucleotide diversity and the number of polymorphic sites were almost identical between the two clades (S3 and S4 Tables). Within- and among-clade distances are represented in S5 Table. The samples apparently originated from the laboratory cultures are listed in S6 Table.

### *COI* phylogenetic tree

**Fig 2:**
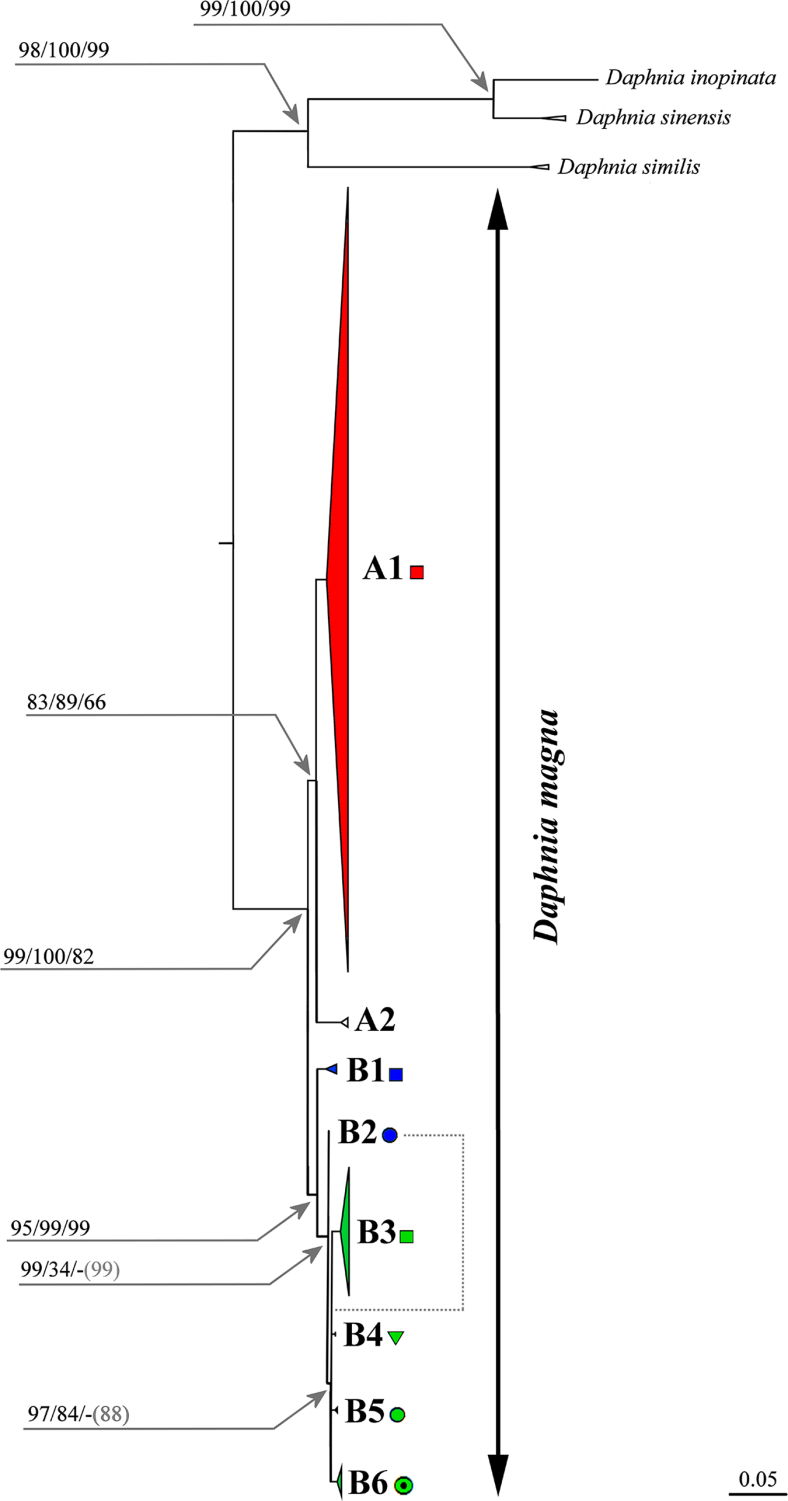
Maximum likelihood tree based on all analyzed *COI*. The support values of individual nodes are based on: Maximum likelihood (ML) / Maximum Parsimony (MP) / Bayesian Inference (BI). Dotted line indicates an incongruence between best-supported topologies using the three methods, with grey numbers indicating BI support for an alternative BI tree topology.

The best-fit model [92] for the whole *COI* data set (our sequences and GenBank sequences combined) was HKY with four categories of gamma (+G4, shape alpha 1.61) and a proportion of invariable sites (+I, 0.602) with a relative BIC weight of 0.3648. Based on the cumulative relative weight, two other models were also within the 95% confidence interval. One best ML tree was found, with an overall likelihood score of -ln L = -2775.1574. These analyses revealed the existence of two highly divergent and well-supported main super-clades within *Dapnia magna* (clades A and B, Fig 2). Each of these two clades was subdivided into several sub-clades Fig 2). Furthermore, the outgroup species formed three clades of the *D*. *similis* group, which were well separated from *D*. *magna*, as already found in previous studies [23,33]. Trees constructed with different methods were congruent with this finding (with a single exception, Fig 2), but the clade support differed among analyses (Fig 2). All deep branches had a strong support in ML and BI. In general, the clades that have a strong support in ML had a weaker support in BI.

The super clade A includes clade A1, which was found in samples from Europe, the Mediterranean region (including North Africa), the Middle East, Turkey and Caucasus, as well as in a few populations in Western Siberia. Within this clade, there are several sequences from the Genbank labeled as originating from North America (Mexico, USA, Canada), but in reality most of these may have originated from laboratory cultures, presumable of European origin (S6 Table). Few others are very likely cases of recent human-mediated invasions (see Discussion). The American individuals with A1 haplotypes were therefore excluded from further analyses and are not represented on any of the figures, except for proven laboratory clones, which are included in S4 Table. The super clade also includes clade A2, which is present exclusively in samples from North America. Unfortunately, there is no exact information on the sampling localities of the sequences deposited to the GenBank, only stated “Manitoba, Canada” [51], and only names of sampling sites (“Round Lake” and “Sue”) with unclear exact geographic locations are provided in [14]. We hence decided to omit all North American samples from the map (Fig 1).

The super-clade B includes several Asian and North American lineages and co-occurs with clade A1 in a single lake in the Novosibirsk Area (Western Siberia) (Fig 1, black arrow). No occurrences of clade A1 were found to the East of this location, nor of super-clade B to the West of this location. Clade B1 is relatively locally distributed in the southern parts of Asian Russia, at the boundary between Western (Ob' basin) and Eastern (Enisey Basin) Siberia. Clade B2 is found in a single water body in North Mongolia. Clade B3 is widely distributed in Eastern Siberia, Japan, and East China. Clade B4 is found in a single water body in Ob' basin (easternmost Western Siberia). Clade B5 is found only in Central Yakutia (Eastern Siberia), and clade B6 is present in Arctic Canada only.

### *COI* median haplotype network

**Fig 3:**
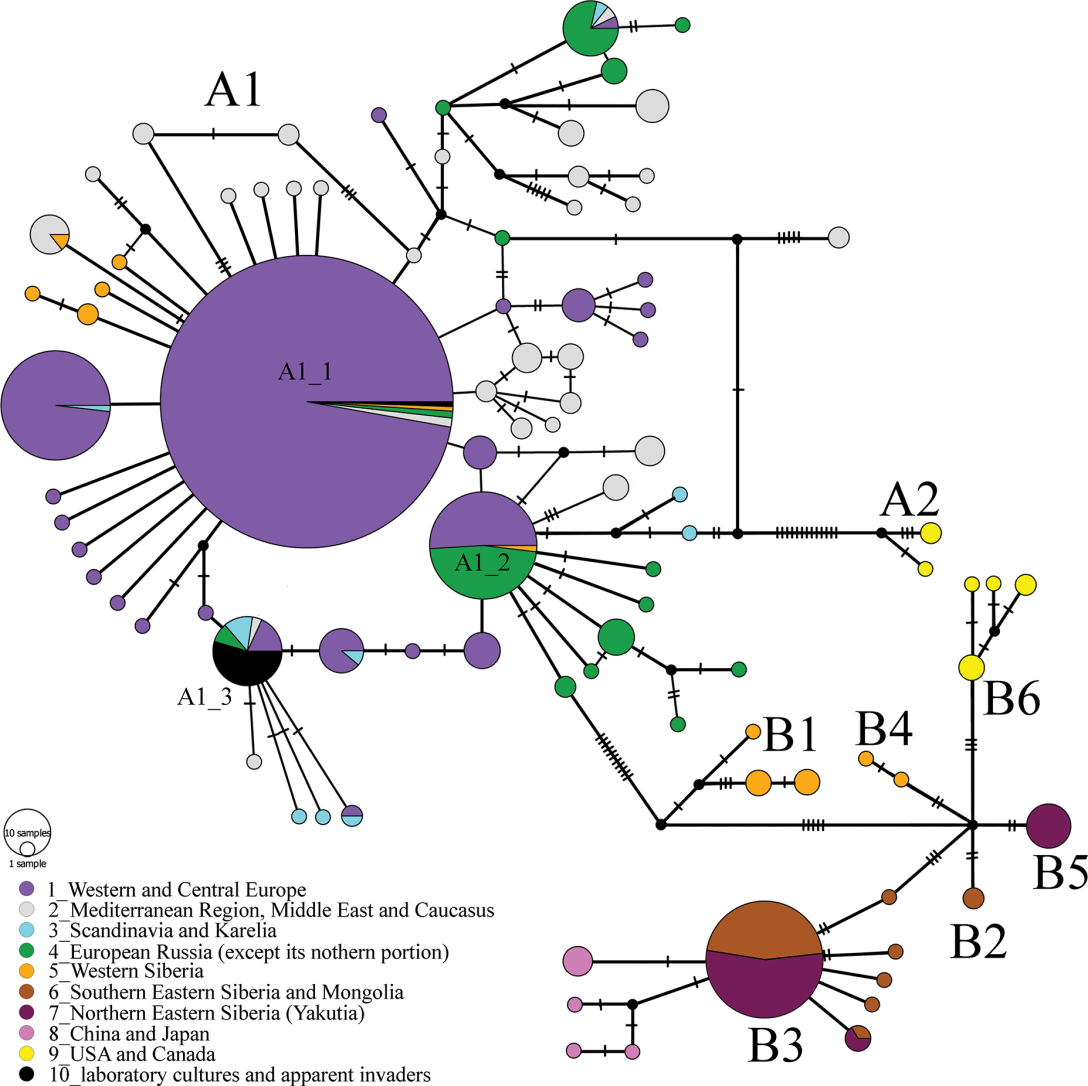
Median-joining *COI* haplotype network. Median vectors are indicated by small black circles.

The TCS network consists of the 93 different *COI* haplotypes (Fig 3). Each of the clades identified in the phylogenetic trees (see above) is well-recognizable in this network.

Super-clade A is again clearly subdivided into a diverse clade A1 and a distant clade A2 (North American samples). Within the A1 clade, we found several sub-networks with a star-like topology, suggesting recent population expansion. The central haplotype of sub-network A1_1 (Fig 3) was found in samples from many European countries, the Mediterranean region, European Russia, and a single population in Western Siberia. The central haplotype of sub-network A1_2 had a similar geographic origin as the major haplotype of A1_1, but was more common in European Russia and was not found in the Mediterranean region nor the Middle East. The central haplotype of sub-network A1_3 again had a similar geographic distribution, but was particularly common among samples from the Middle East.

Among the super-clade B, the only clade B3 showed a star-like topology, with its central haplotype being found in samples from Mongolia, China, and several areas of Eastern Siberia (Yakutia, Zabaikalsky Territory, Irkutsk Area), but not penetrating to the west of Lake Baikal. The clade B1 is separated from others by five mutations. All other clades (B2, B3, B4, B5, B6) form a remarkable monophyletic group interconnected through a hypothetical (extinct or un-sampled) haplotype. Tajima's *D* and mismatch distribution analyses (S4 Table) were consistent with the hypothesis of recent population expansion for all these clades, except B6.

### Nested clade phylogeographic analysis

The maximum parsimony network of the major *COI* clades of *D*. *magna* (S1 Fig) shows the two main clades of the highest rank, corresponding to super-clades A and B, to be clearly separated, with demographic processes (sensu Templeton 2004) of the long-distance colonization type (S4 Table). In the European-Western Siberian clade A1, we can see a complicated network with several equally probable topologies. Separation of the clade B6 from North America is likely explained by isolation by distance (S4 Table).

### bGMYC

The results of the bMYGC analysis (S2 Fig) provide no strong support for separate species (i.e., no populations or groups of populations from geographically close localities with posterior probabilities of conspecifity of < 0.05). However, the deep branches are well-supported, especially between super-clades A and B, which have a posterior probability of conspecifity of less than 0.1 (though > 0.05). Within the super-clade A, the European-Western Siberian clade A1 and the North American clade A2 are differentiated. The internal structure of the super-clade B is more complicated: it contains at least three sub-groups: A Western Siberian group (s34, 35, 36) corresponding to clade B1 of the TCS network, an Eastern Siberian-Far Eastern group (s44, 45, 46) corresponding to clades B2-B5 of the network, and a Canadian group (s40, 41, 42) corresponding to clade B6. Therefore, this analysis confirms the complexity of the *D*. *magna* phylogeographic structure, especially in the Asian Palaearctic region. Furthermore, the high genetic diversity of *D*. *magna* is confirmed by the *p*-distances, which range up to >5%. At the same time, outgroup species differ from any clades within *D*. *magna* by 14–17%.

### ABGD test

The ABDG test based on haplotype groups (s_numbers) according to the star contraction algorithm [80] revealed the following large groups within *D*. *magna*: Group 1 –Eastern Siberian clade B3 (s44, 45, 46), Group 2 –Eastern Siberian clades B2, B4, B5 (s37, 38, 39, 43), Group 3—Western Siberian clade B1 (s34, 35, 36), Group 4 –Canadian clade B6 (s40, 41, 42), Group 5 –North American clade A2 (s17, 18), and Group 6 –European clade A1.

### Divergence time estimates

The divergence time estimates differed strongly, depending on the method used. Following the procedures and calibrations of [90], we obtained divergence time estimates between clades A and B of 2.2–2.8 MYA, 2.5 MY between clades A1 and A2, 2.6 MY between the Canadian B6 and other B-subclades, and 10–11 MY between D. *magna* and the outgroup species. However, using the additional calibration of the sub-genera *Ctenodaphnia* / *Daphni*a split, which was estimated at 145 MYA [91], resulted in almost ten times larger divergence time estimates, 17–20 MY between clades A and B, 15 MY between A1 and A2, 22 MY between B6 and the other B-subclades, and 100–120 MY between *D*. *mag*na and other *Ctenodaphnia* species (S5 Table).

### Results on one additional mitochondrial and three nuclear gene fragments

As mentioned above, the results on the other gene fragments were inconclusive and therefore only presented in S1 and S3 Tables, S1 Text and S3 and S4 Figs.

## Discussion

### Phylogeography: General patterns

Our analyses consistently and unambiguously identified a deep split between Eastern and Western mitochondrial haplotypes of *D*. *magna* in Eurasia, in particular between the European-Middle Eastern-Western Siberian = Western Eurasian clade (A1) and the Eastern Siberian-Far Eastern-Chinese-"Beringian" = East Asian ones (B1-B5). Sampling locations were distributed almost evenly from Western Europe to Easternmost Asia, and the transition between the two groups appears to be rather abrupt. The network structure suggests that this suture zone (sensu [93–95]) is a secondary contact zone between the two super-clades. The width of this zone and its North-South extension cannot currently be assessed due to a lack of available samples. However, the lake in which the two super-clades are found to co-occur is located in the Ob' River basin, in the Easternmost part of Western Siberia, somewhat west to the transition zone between two super-groups of haplotypes previously identified in other cladocerans [31,32]. Regardless, our findings provide strong additional evidence for a marked longitudinal (East-West) differentiation of the cladoceran fauna in Northern Eurasia.

### Phylogeography: Local patterns of mitochondrial haplotype distributions

On a more local scale, we find little evidence for a strong geographic structure of mitochondrial haplotypes within the Western A1 clade, consistent with previous studies [14]. The main A1 subclades show a wide geographic distribution, and co-occur in different regions, though at different frequencies. Together with the evidence for relatively recent population expansion, a possible scenario is expansion from several refugia and subsequent mixing through migration. The current data do not allow us to identify the location of these refugia, though the different frequencies of the subclades in different regions may provide some indications. Haplotype diversity is especially high in the Mediterranean region and the Middle East and involves many peripheral haplotypes in the star-like network. These might be relicts of a pre-Pleistocene diversity of the clade A1, with only a subset of this diversity being involved in recent population expansion.

The individual clades within super-clade B are more distinct, though the existence of intermediate haplotypes in non-sampled populations remains a realistic possibility. Several clades (B1, B2, B4, B5) were found only in a small region or even only at a single location, either at the boundary between Eastern and Western Siberia (B1, B2, B4) or in Northeastern Siberia (B5). All aforementioned B clades may be relicts of a previous, hypothetical "pan-Beringian" population (East Asia plus North America, with no evidence for contemporary occurrence of *D*. *magna* in easternmost Siberia nor westernmost North America). Interestingly, the clade B1, which is the clade that occurs in the contact zone between A and B super-clades, has an intermediate position between the two super-clades in the haplotype network (though it clearly belongs to super-clade B, and hence has a more recent common ancestor with other B clades than with A clades). The interpretation of this observation needs further investigation, but again highlights the special interest of the boundary between Western and Eastern Siberia.

The super clade B also contains the more widespread clade B3, distributed across large parts of Eastern Siberia, Northeastern Siberia, as well as the Far East, with evidence for recent population expansion. The fact that haplotypes from China and Japan do not belong to the central group of the B3 clade, but are offshoots of it, suggests that these more southern regions may have been colonized from the north (Eastern Siberia, Mongolia), in contrast to the more common northward expansion that is assumed for most postglacial colonization events [32].

Two clades A2 and B6 were found exclusively in North America. We will discuss the phylogeography of *D*. *magna* in North America only very briefly here, as it is clear that too few samples from that continent were available for our study to reach more detailed conclusions. Furthermore, the precise sampling locations of this material are unclear (see above). Nonetheless, the observation that both super-clades seem to be present in North America, with haplotypes that are clearly distinct from the haplotype clades found in Europe and Asia, suggests the possibility that North America may historically have been colonized from both sides (for evidence of more recent colonization, see below).

We have used two common approaches to separate evolutionary significant groups, the Nested Clade Phylogeographical Analysis [96,83] and a Bayesian approach realized in the GMYC model [75]. The NCPA has been widely used in cladoceran phylogeography [14,27,97], but its use has been criticized [98] and its results must be discussed with a great care. In most cases, separation of the highest rank of clades in NCPA is unproblematic, but the adequateness of lower-rank clades is questionable, despite the reduction of phylogenetic noise with the star contraction algorithm [80]. In our case, the NCPA suggested for almost all groups of *D*. *magna* a good fit with the RGF (restricted gene flow) model, which is in agreement with the cladoceran biology (existence of isolated populations with a large number of generations per season). For the high-ranking super-clades (A and B) the finding of correspondence to the RGF model is a useful conclusion, as it suggests long-term (but potentially incomplete) isolation.

The bGMYC, on the other hand, is very sensitive to the mathematical model parameters [83], but apparently gives better results for long-term phylogenetic reconstructions [75]. The considerable *COI* polymorphism in *D*. *magna* allowed us to use this method here, with apparently more adequate conclusions concerning larger clades. In the literature, methods of statistical analysis of phylogeographic patterns appear to be chosen somewhat haphazardly, rather than according to the advantages and limits of each method for a given data set (see [83,98]). Moreover, complicated models are often used, which are too sensitive to their parameters [63]. We therefore think that results of sophisticated methods must be interpreted with a great care, and should be verified with independent data and/or alternative methods. Moreover, simpler methods, such as the separation of evolutionary significant units according to genetic distance [63] may be preferable in some cases, compared to more sophisticated ones [99]. Therefore, the application of algorithms of phylogeographic reconstructions should be accompanied by a critical analysis of the results, taking into account species biology.

### No evidence for cryptic species

Many previous genetic studies have advocated the existence of species complexes instead of single species among different species groups of *Daphnia* (see [9,12]). Although we detected two highly distinct mitochondrial super-clades within *Daphnia magna*, which may be interpreted as separate taxa (e.g., by the ABGD test), several lines of evidence strongly suggest that they represent two divergent super-clades within a single species. First, the genetic distance between the two super clades is much smaller than between *D*. *similis*, *D*. *sinensis*, and *D*. *inopinata*, which are good, though very closely related biological species with reproductive isolation strongly supported by differences in male morphology [33]. Second, no concordant subdivision was observed in nuclear genes (S1 Text, though this may be due to a lack of sufficient variability of these genes), and, third, and most importantly, individuals from the two super-clades readily interbreed, at least in the laboratory [100]. Therefore, to date there is no evidence of the existence of cryptic species within *D*. *magna*.

### Invasions

Cladocerans, and particularly some species of *Daphnia*, are very popular laboratory animals and are often used as food in aquaculture. Moreover, their resting stages are long-lived, which favors dispersal by human activities (e.g., the ephippia of *D*. *magna* of super-clade A were found in ballast water [56]). Many *COI* sequences of *D*. *magna* are marked on GenBank as belonging to "Mexican" or "Canadian" populations (S6 Table). However, closer investigation led to the conclusion that these contain many specimens that originated from laboratory cultures in those countries. The genetic variability among specimens with known laboratory origin (“cultures” in S6 Table) is very low. Possibly, all these specimens derive from commercial clones (or perhaps from a single clone) from Aquatic BioSystems Inc., Fort Collins, Colorado, USA, which ships ephippia to customers. Likely, one of these clones was introduced to the lake near Dorset Environmental Science Center, Ontario (as it has the same haplotype as the "cultures"). Therefore we hypothesize that only clades A2 and B6 represent specimens of true North American origin, whereas North American specimens belonging to A1 are likely explained by anthropogenic invasion.

In general, *D*. *magna* does not seems to be a species with a strong invasive potential–in contrast to *D*. *lumholtzi*, *D*. cf. *pulex*, and *D*. *galeata* [101–103]. But recent anthropogenic invasions are revealed here, at least of the clade A1 probably in North America. Such anthropogenic invasions could strongly affect phylogeograpic conclusions. For example, it is possible that the distributional ranges of the three central haplotypes in Europe have been strongly enlarged by human activities. Therefore invasions (both detected and undetected ones) complicate phylogeographic analyses of *Daphnia* and many other organisms, and require a critical interpretation of the obtained results.

### Molecular clock and dating of divergence times

The two molecular clock scenarios used yielded strongly different results on the timing of divergence of *D*. *magna* from its sister species, as well as of the super-clades and clades within the species. Currently the issue remains unresolved, and its resolution will have to take into account Frey's [6,7] paradigm of the cladoceran biogeography, morphological stasis during the last millions of year, up to ten-fold differences in evolutionary rates between sister clades [104], as well as the possibility of decreasing mutation rates as a function of time [105,106]. In the end, it seems that the question can only be resolved with appropriate palaeontological records, which are, unfortunately, still far too rare for pre-Pleistocene cladocerans [107].

## Supporting information

S1 Fig Maximum parsimony *COI* network and nested clade design of major clades of *D*. *magna*. **Numbers identify star groups of haplotypes (see S2 Table).** Circle size is proportional to the frequency of the haplotypes. Small dark circles indicate unsampled or extinct haplotypes. Geographic regions for star groups are as follows: s1 –Western, Central Europe, Mediterranean region, Middle East; s2 –Western Europe; s3 –Scandinavian Peninsula and Western Europe; s4-s8 –Mediterranean region; s9 –Mediterranean region and Near East; s10 –Western Europe; s11 –European Russia; s12—Near East; s13-s14 –European Russia; s15-s16 –Mediterranean region; s17-s18 –North America; s19 –European Russia s20 –Mediterranean region; s21 –Western Europe; s22 –Western and Central Europe; s23 –Central and Eastern Europe; s24-s25 –Western Europe; s26 –Western Europe and Scandinavian Peninsula; s27 –different regions of Europe and laboratory cultures; s28 –Mediterranean region; s29 –Western and Central Europe; s30 –Scandinavian Peninsula; s31-s33 –European Russia; s34-s36 –Western Siberia; s37 –Eastern Siberia; s38-s39 –Western Siberia; s40-s42 –North America; s43-s45 –Eastern Siberia; s46 –Far East of Asia.
(TIF)

S2 Fig Maximum clade credibility tree for *COI* as identified with the bGMYC analysis. **Clades highlighted in red represent the maximum likelihood limits.** The colored lines correspond to a sequence-by-sequence matrix, with lines colored according to the posterior probability that the sequences are conspecific, which allows visualizing uncertainty in group limits.
(TIF)

S3 Fig Bayesian tree based on concatenated mitochondrial *16s* rRNA sequences and nuclear *18s* rRNA sequences (1036 bp). All original samples have prefix zh, while the Genbank samples have no this prefix. GenBank accession numbers for original samples are given in S1 Table, for sequences obtained from GenBank they are: USA–AY921452, Denmark–DQ470575, Belgium–AM490278, China1 –KF993366, China2 –KM244710, China3 –KP296147, China4 –NC_026914, Novosibirsk1 –JN874603, Novosibirsk2 –JN874602, Novosibirsk3 –JN874604, Novosibirsk4 –JN874601.
(TIF)

S4 Fig Bayesian tree based on concatenated nuclear loci of *HSP90* and histone *H3* sequences (994 bp). All original samples have prefix zh, while the Genbank samples do not have this prefix. GenBank accession numbers for original samples are given in S1 Table, for sequences obtained from GenBank they are: USA—DQ845268.
(PDF)

S1 Table Complete list of original sequences obtained in this study with information on sampling localities and the GenBank accession numbers for *COI*, *16S*, *18S*, *HSP*, *H3* sequences for each specimen. *COI* clade designations as defined in Fig 2.
(XLS)

S2 Table Original and GenBank *COI* sequences for *Dapnia magna* arranged according to haplotype clades, star-groups, and geographic regions. (XLS)

S3 Table Genetic diversity of *Daphnia* species. n—sample size, Nh—number of haplotypes, Nv—number of variable (polymorphic) sites, Np—number of parsimony informative sites, Hd—haplotype diversity, Pi—nucleotide diversity, k—average number of nucleotide differences.
(DOC)

S4 Table Polymorphism of the *COI* gene mtDNA of the *Daphnia magna* populations. n—sample size, Nh—number of haplotypes, Nv—number of variable (polymorphic) sites, Np—number of parsimony informative sites, Hd—haplotype diversity, Pi—nucleotide diversity, k—average number of nucleotide differences; R2 population size expansion test and results of Tajima’s D, Fu’s FS and mismatch distributions: tau-parameter, SSD (sum of squares deviation) and Harpending's Raggedness index including associated p-values. Biological processes are (Templeton, 2004): RGF–restricted gene flow; D–dispersal; LDD–long-distance dispersal; IBD–isolation by distance; AF–allopatric fragmentation; PF–past fragmentation; LDC–long-distance colonization.
(DOC)

S5 Table Between-group *p*-distances (in percent) among the *D*. *magna* population’s groups for *COI* gene. In diagonal, bold–estimates of average evolutionary divergence over sequence pairs within groups. Below diagonal–estimates of evolutionary divergence over sequence pairs between groups. The presence of n/c in the results denotes cases in which it was not possible to estimate evolutionary distances. Above diagonal–time divergence, in MYA; first digit–“fast clock” from (Schwentner *et al*., 2013), second digit–“slow clock” from (Kotov & Taylor, 2011).
(DOC)

S6 Table Real sources of some *COI* sequences represented in the GenBank. (DOC)

S1 Text Results on one additional mitochondrial and three nuclear gene fragments. (DOCX)
