# Supplementary figures and images for: Phylogeography of Daphnia magna Straus (Crustacea: Cladocera) in Northern Eurasia: Evidence for a deep longitudinal split between mitochondrial lineages
Source: PLoS One. 2018 Mar 15;13(3):e0194045. doi: 10.1371/journal.pone.0194045 (PMC5854346; doi:10.1371/journal.pone.0194045)

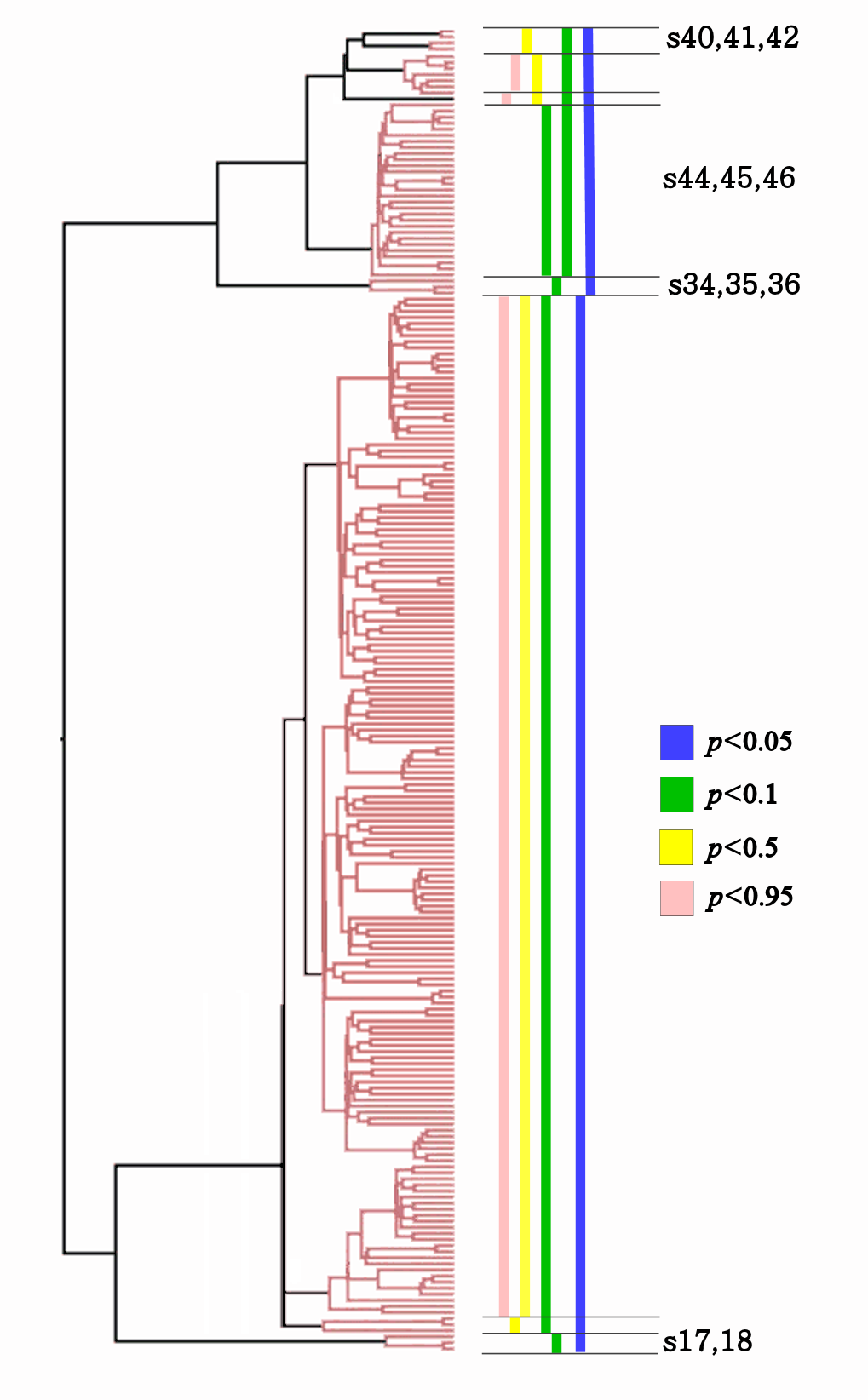

Supplement: S1 Fig — Numbers identify star groups of haplotypes (see S2 Table). Circle size is proportional to the frequency of the haplotypes. Small dark circles indicate unsampled or extinct haplotypes. Geographic regions for star groups are as follows: s1 –Western, Central Europe, Mediterranean region, Middle East; s2 –Western Europe; s3 –Scandinavian Peninsula and Western Europe; s4-s8 –Mediterranean region; s9 –Mediterranean region and Near East; s10 –Western Europe; s11 –European Russia; s12—Near East; s13-s14 –European Russia; s15-s16 –Mediterranean region; s17-s18 –North America; s19 –European Russia s20 –Mediterranean region; s21 –Western Europe; s22 –Western and Central Europe; s23 –Central and Eastern Europe; s24-s25 –Western Europe; s26 –Western Europe and Scandinavian Peninsula; s27 –different regions of Europe and laboratory cultures; s28 –Mediterranean region; s29 –Western and Central Europe; s30 –Scandinavian Peninsula; s31-s33 –European Russia; s34-s36 –Western Siberia; s37 –Eastern Siberia; s38-s39 –Western Siberia; s40-s42 –North America; s43-s45 –Eastern Siberia; s46 –Far East of Asia. (TIF) [file pone.0194045.s001.tif]

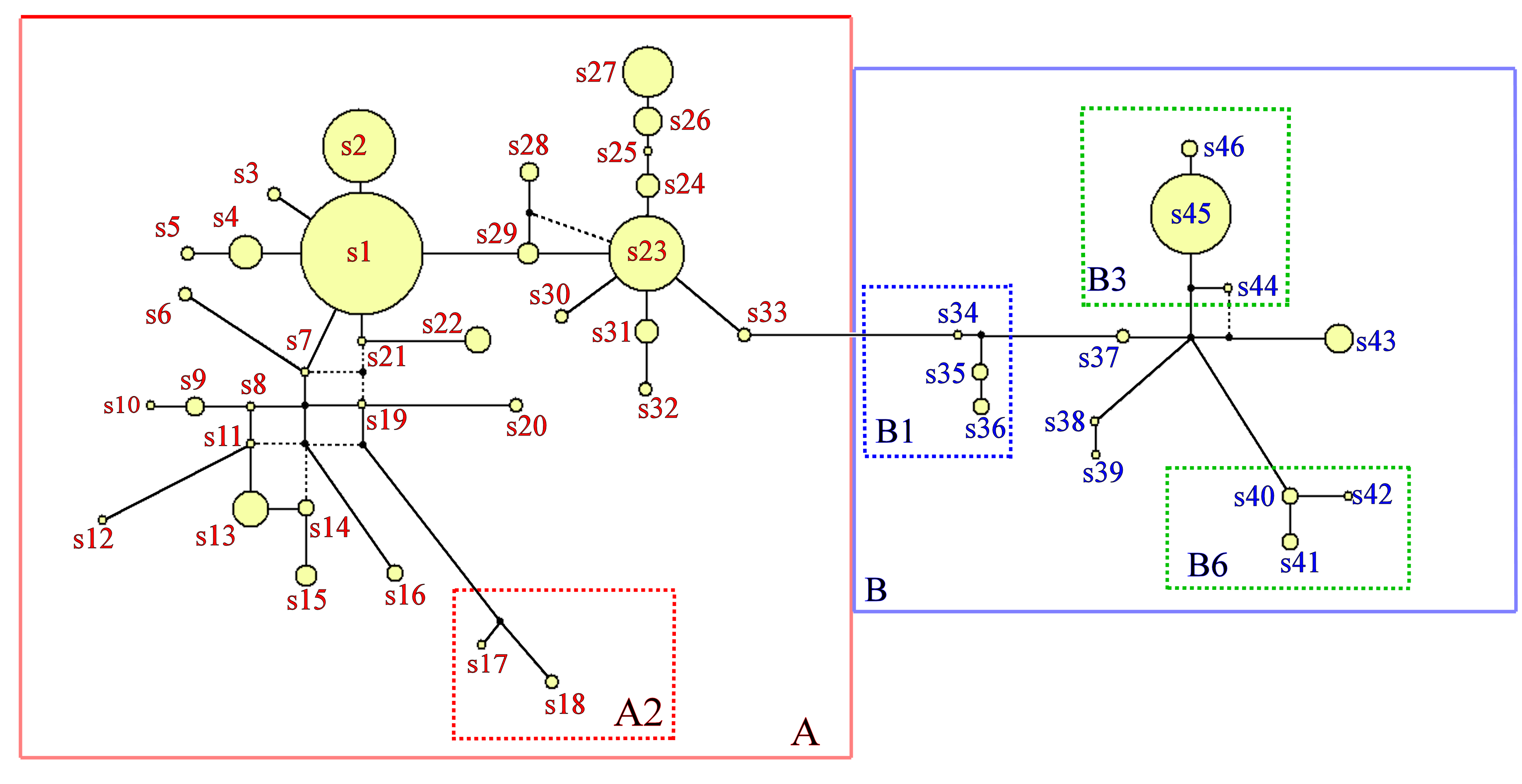

Supplement: S2 Fig — Clades highlighted in red represent the maximum likelihood limits. The colored lines correspond to a sequence-by-sequence matrix, with lines colored according to the posterior probability that the sequences are conspecific, which allows visualizing uncertainty in group limits. (TIF) [file pone.0194045.s002.tif]

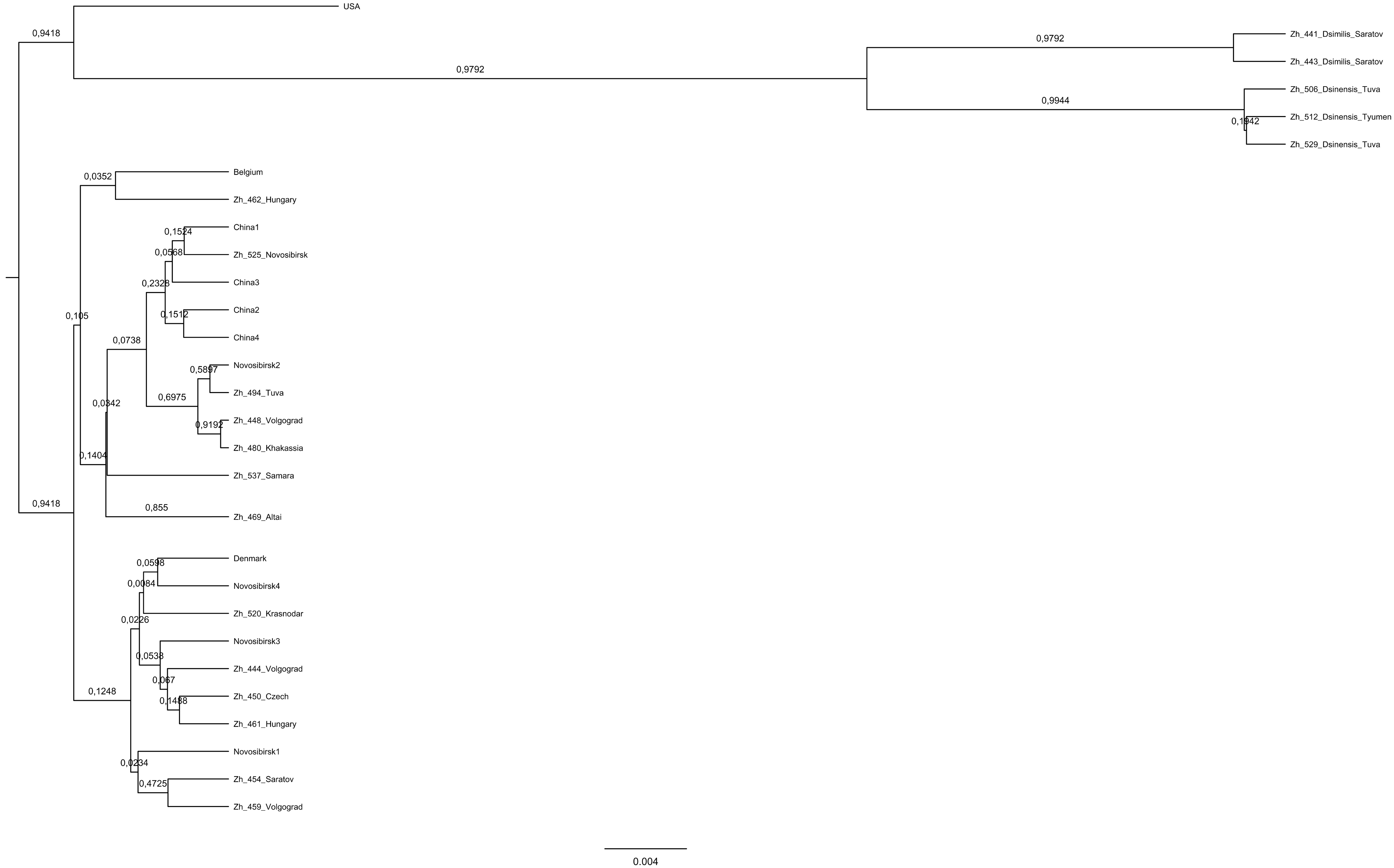

Supplement: S3 Fig — All original samples have prefix zh, while the Genbank samples have no this prefix. GenBank accession numbers for original samples are given in S1 Table, for sequences obtained from GenBank they are: USA–AY921452, Denmark–DQ470575, Belgium–AM490278, China1 –KF993366, China2 –KM244710, China3 –KP296147, China4 –NC_026914, Novosibirsk1 –JN874603, Novosibirsk2 –JN874602, Novosibirsk3 –JN874604, Novosibirsk4 –JN874601. (TIF) [file pone.0194045.s003.tif]

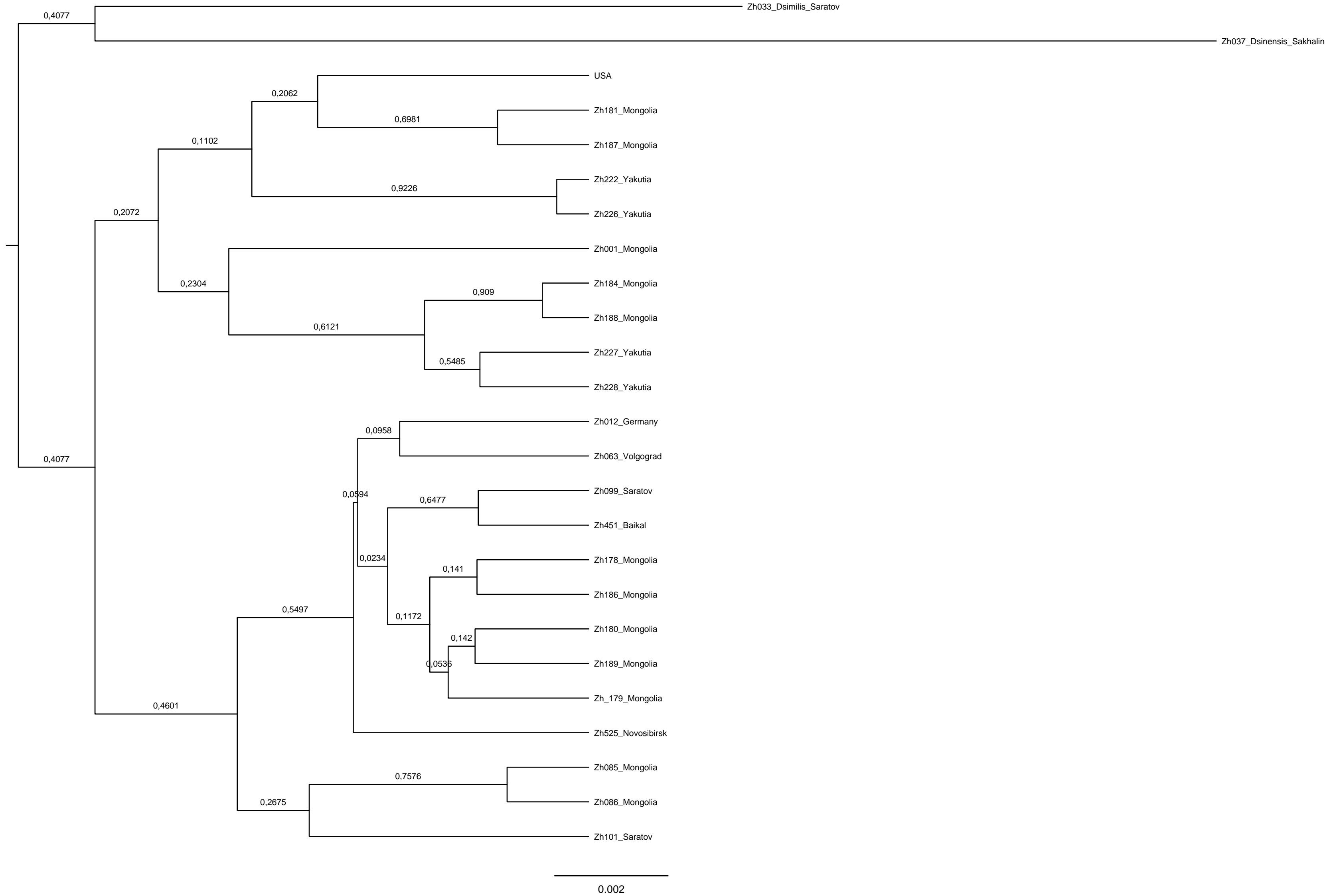

Supplement: S4 Fig — All original samples have prefix zh, while the Genbank samples do not have this prefix. GenBank accession numbers for original samples are given in S1 Table, for sequences obtained from GenBank they are: USA—DQ845268. (PDF) [file pone.0194045.s004.pdf]
