# Supplementary material for: Phylogeography of Daphnia magna Straus (Crustacea: Cladocera) in Northern Eurasia: Evidence for a deep longitudinal split between mitochondrial lineages
Source: PLoS One. 2018 Mar 15;13(3):e0194045. doi: 10.1371/journal.pone.0194045 (PMC5854346; doi:10.1371/journal.pone.0194045)
Supplement: S3 Table — n—sample size, Nh—number of haplotypes, Nv—number of variable (polymorphic) sites, Np—number of parsimony informative sites, Hd—haplotype diversity, Pi—nucleotide diversity, k—average number of nucleotide differences. (DOC) [file pone.0194045.s007.doc]

**S3 Table. Genetic diversity and GenBank accession numbers of original sequences presented in this study.** n - sample size, Nh - number of haplotypes, Nv - number of variable (polymorphic) sites, Np - number of parsimony informative sites, Hd - haplotype diversity, Pi - nucleotide diversity, k - average number of nucleotide differences.

| Species | Fragment | Super-Clade | Fragment length | n | Nh | Nv | Np | Hd | Pi | k | GenBank  accession Nos. |
| --- | --- | --- | --- | --- | --- | --- | --- | --- | --- | --- | --- |
| *D. magna* | COI | A | 563 bp  (563-668) | 72 | 21 | 28 | 17 | 0.855 | 0.006 | 3.4 | MF346401-MF346479 |
| *D. magna* | COI | B | 563 bp  (563-668) | 83 | 15 | 29 | 22 | 0.556 | 0.007 | 4.2 |
| *D. magna* | COI | total | 563 bp  (563-668) | 155 | 36 | 56 | 44 | 0.840 | 0.012 | 12.6 |
| *D. magna* | 16S |  | 450 bp | 12 | 10 | 9 | 7 | 0.949 | 0.008 | 3.6 | MF346498, MF346501-MF346511 |
| *D. magna* | 18S |  | 585 bp | 11 | 2 | 3 | 0 | 0.154 | 0.001 | 0.5 | MF346517-MF346527 |
| *D. magna* | HSP-90 |  | 587 bp | 18 | 12 | 13 | 10 | 0.924 | 0.005 | 3.2 | MF346481-MF346492 |
| *D. magna* | H3 |  | 395 bp | 3 | 3 | 11 | 0 | - | 0.018 | 7.3 | MF346495- MF346497 |
| *D. similis* | COI |  | 668 bp | 3 | 2 | 9 | 9 | - | 0.009 | 5.4 | MF346399-MF346400 |
| *D. similis* | 18S |  | 586 bp | 2 | 1 | 0 | 0 | - | - | - | MF346512-MF346513 |
| *D. similis* | H3 |  | 395 bp | 1 | 1 | 0 | 0 | - | - | - | MF346493 |
| *D. sinensis* | COI |  | 668 bp | 16 | 7 | 18 | 8 | 0.637 | 0.004 | 2.4 | MF346385-MF346398 |
| *D. sinensis* | 16S |  | 448 bp | 2 | 1 | 0 | 0 | - | - | - | MF346499-MF346500 |
| *D. sinensis* | 18S |  | 585 bp | 3 | 1 | 0 | 0 | - | - | - | MF346514-MF346515 |
| *D. sinensis* | HSP-90 |  | 587 bp | 1 | 1 | - | - | - | - | - | MF346480 |
| *D. sinensis* | H3 |  | 395 bp | 1 | 1 | - | - | - | - | - | MF346494 |
