# Supplementary material for: Phylogeography of Daphnia magna Straus (Crustacea: Cladocera) in Northern Eurasia: Evidence for a deep longitudinal split between mitochondrial lineages
Source: PLoS One. 2018 Mar 15;13(3):e0194045. doi: 10.1371/journal.pone.0194045 (PMC5854346; doi:10.1371/journal.pone.0194045)
Supplement: S4 Table — n—sample size, Nh—number of haplotypes, Nv—number of variable (polymorphic) sites, Np—number of parsimony informative sites, Hd—haplotype diversity, Pi—nucleotide diversity, k—average number of nucleotide differences; R2 population size expansion test and results of Tajima’s D, Fu’s FS and mismatch distributions: tau-parameter, SSD (sum of squares deviation) and Harpending's Raggedness index including associated p-values. Biological processes are (Templeton, 2004): RGF–restricted gene flow; D–dispersal; LDD–long-distance dispersal; IBD–isolation by distance; AF–allopatric fragmentation; PF–past fragmentation; LDC–long-distance colonization. (DOC) [file pone.0194045.s008.doc]

**S4 Table. Polymorphism of the COI fragment in the different *Daphnia magna* clades (GenBank and original data combined).** n - sample size, Nh - number of haplotypes, Nv - number of variable (polymorphic) sites, Np - number of parsimony informative sites, Hd - haplotype diversity, Pi - nucleotide diversity, k - average number of nucleotide differences; R2 population size expansion test and results of Tajima’s D, Fu’s FS and mismatch distributions: tau-parameter, SSD (sum of squares deviation) and Harpending's Raggedness index including associated *p*-values. Biological processes are (Templeton, 2004): RGF – restricted gene flow; D – dispersal; LDD – long-distance dispersal; IBD – isolation by distance; AF – allopatric fragmentation; PF – past fragmentation; LDC – long-distance colonization. “Сultures” refers to the subset of samples that are apparently originated from the laboratory clones.

| Clade | n | Nh | Np | Hd | Pi | Tajima’s D | Fu’s FS | R2-test | Mismatch distributions | | | Population  process |
| --- | --- | --- | --- | --- | --- | --- | --- | --- | --- | --- | --- | --- |
|  |  |  |  |  |  | D  (*p*) | FS  (*p*) | R2  (*p*) | Tau | SSD  (*P_SSD_*) | R  (*P_R_*) |  |
| A1 | 648 | 68 | 35 | 0.618 | 0.003 | -2.201  (<0.05) | -26.655  (<0.05) | 0.015  (<0.05) | 0.001 | 0.007  (0.8) | 0.054  (0.64) | RGF/D/LDD |
| A2 | 3 | 2 | 0 | 0.667 | 0.004 | - | - | - | - | - | - | - |
| B1 | 2 | 1 | 0 | - | - | - | - | - | - | - | - | - |
| B2 | 2 | 1 | 0 | - | - | - | - | - | - | - | - | - |
| B3 | 72 | 10 | 4 | 0.035 | 0.001 | -2.172  (<0.05) | -8.41  (<0.05) | 0.035  (<0.05) | 1.04 | 0.001  (0.61) | 0.214  (0.78) | RGF/LLD |
| B4 | 2 | 2 | 0 | - | - | - | - | - | - | - | - | - |
| B5 | 9 | 1 | 0 | - | - | - | - | - | - | - | - | - |
| B6 | 7 | 4 | 2 | 0.81 | 0.003 | -0.039  (>0.1) | -0.583  (>0.1) | 0.172  (>0.1) | 2.15 | 0.08  (0.14) | 0.31  (0.26) | IBD |
| cultures | 14 | 2 | 4 | 0.264 | 0.002 | -0.531  (>0.1) | 2.697  (<0.1) | 0.131  (<0.1) | 4.572 | 0.047  (0.24) | 0.68  (0.71) | - |
| Super-clade A | 651 | 70 | 47 | 0.621 | 0.004 | -2.275  (<0.05) | -26.661  (<0.05) | 0.013  (<0.05) | 0.001 | 0.007  (0.79) | 0.053  (0.65) | AF/LDC |
| Super-clade B | 99 | 21 | 29 | 0.648 | 0.008 | -0.936  (>0.1) | -25.585  (<0.05) | 0.066  (<0.05) | 7.577 | 0.017  (0.87) | 0.098  (0.81) | LDC |
| Total | 764 | 93 | 70 | 0.716 | 0.012 | -1.429  (<0.05) | -24.178  (<0.05) | 0.034  (<0.05) | 22.158 | 0.022  (0.90) | 0.039  (0.88) | PF/LDC |
