# Supplementary material for: Phylogeography of Daphnia magna Straus (Crustacea: Cladocera) in Northern Eurasia: Evidence for a deep longitudinal split between mitochondrial lineages
Source: PLoS One. 2018 Mar 15;13(3):e0194045. doi: 10.1371/journal.pone.0194045 (PMC5854346; doi:10.1371/journal.pone.0194045)
Supplement: S5 Table — In diagonal, bold–estimates of average evolutionary divergence over sequence pairs within groups. Below diagonal–estimates of evolutionary divergence over sequence pairs between groups. The presence of n/c in the results denotes cases in which it was not possible to estimate evolutionary distances. Above diagonal–time divergence, in MYA; first digit–“fast clock” from (Schwentner et al., 2013), second digit–“slow clock” from (Kotov & Taylor, 2011). (DOC) [file pone.0194045.s009.doc]

**S5 Table. Between-group *p*-distances (in percent) among the different species and *D. magna* clades for the COI gene.** Diagonal (bold face) – estimates of average evolutionary distance between pairs of sequences within groups (n/c indicates that it was not possible to estimate the evolutionary distances). Below diagonal – estimates of evolutionary distance between pairs of sequences between groups. Above diagonal –divergence time, in MYA; first number – “fast clock” from (Schwentn*er et al.*, 2013), second number – “slow clock” from (Kotov & Taylor, 2011).

|  | A1 | A2 | B1 | B2 | B3 | B4 | B5 | B6 | *D. inopinata* | *D. similis* | *D. sinensis* |
| --- | --- | --- | --- | --- | --- | --- | --- | --- | --- | --- | --- |
| A1 | **0.335** | 2.5/24.8 | 2.3/22.2 | 2.4/23.4 | 2.8/27.1 | 2.8/27.5 | 2.9/28.1 | 2.9/28.3 | 11.8/116.7 | 10.7/106.1 | 10.8/106.9 |
| A2 | 3.596 | **0.474** | 3.2/31.2 | 3.0/29.8 | 3.4/33.6 | 3.5/34.1 | 2.9/34.8 | 3.7/36.9 | 10.9/107.1 | 10.2/100.6 | 10.1/99.7 |
| B1 | 3.221 | 4.525 | **n/c** | 1.4/13.7 | 1.8/17.6 | 1.8/18.0 | 1.9/18.6 | 1.9/18.6 | 11.7/115.6 | 11.1/109.7 | 10.7/105.2 |
| B2 | 3.395 | 4.322 | 1.979 | **n/c** | 0.9/8.9 | 0.6/5.5 | 0.5/4.9 | 0.7/7.4 | 12.1/119.0 | 10.8/106.3 | 10.7/105.5 |
| B3 | 3.931 | 4.870 | 2.542 | 1.288 | **0.097** | 1.0/9.5 | 0.9/8.9 | 1.1/11.2 | 11.9/117.5 | 11.3/111.2 | 10.7/105.8 |
| B4 | 3.988 | 4.944 | 2.601 | 0.799 | 1.377 | **0.178** | 0.6/5.5 | 0.8/8.0 | 12.0/118.3 | 11.1/109.4 | 10.6/104.9 |
| B5 | 4.083 | 5.033 | 2.690 | 0.710 | 1.283 | 0.799 | **n/c** | 0.7/7.4 | 11.8/116.5 | 10.7/105.1 | 10.6/104.7 |
| B6 | 4.099 | 5.337 | 2.690 | 1.066 | 1.627 | 1.155 | 1.066 | **0.288** | 12.0/117.9 | 10.9/107.4 | 10.5/103.1 |
| *D. inopinata* | 16.908 | 15.512 | 16.747 | 17.229 | 17.024 | 17.140 | 16.874 | 17.077 | **n/c** | 10.5/103.1 | 6.1/60.6 |
| *D. similis* | 15.351 | 14.565 | 15.892 | 15.402 | 16.100 | 15.846 | 15.225 | 15.554 | 14.945 | **1.277** | 11.1/101.8 |
| *D. sinensis* | 15.480 | 14.446 | 15.235 | 15.275 | 15.318 | 15.199 | 15.159 | 14.932 | 8.777 | 15.656 | **0.406** |
