# Supplementary material for: Phylogeography of Daphnia magna Straus (Crustacea: Cladocera) in Northern Eurasia: Evidence for a deep longitudinal split between mitochondrial lineages
Source: PLoS One. 2018 Mar 15;13(3):e0194045. doi: 10.1371/journal.pone.0194045 (PMC5854346; doi:10.1371/journal.pone.0194045)
Supplement: S6 Table — (DOC) [file pone.0194045.s010.doc]

**S6 Table**. Likely sources of some of the *COI* sequences published in GenBank.

| **GenBank number** | **Country according to the GenBank record** | **Likely source of specimen** | **Comment** |
| --- | --- | --- | --- |
| EU702134 | Mexico | Ecotoxicology Laboratory IPN, Mexico City | Apparently a laboratory culture |
| EU702138 | Mexico | Ecotoxicology Laboratory IPN, Mexico City | Apparently a laboratory culture |
| EU702137 | Mexico | Ecotoxicology Laboratory IPN, Mexico City | Apparently a laboratory culture |
| EU702136 | Mexico | Ecotoxicology Laboratory IPN, Mexico City | Apparently a laboratory culture |
| KC616973 | Mexico | Instituto Mexicano de Tecnologia del Agua, Morelos | Most probably, a laboratory clone |
| KC616975 | Mexico | Instituto Mexicano de Tecnologia del Agua, Morelos | Most probably, a laboratory clone |
| KC616976 | Mexico | Instituto Mexicano de Tecnologia del Agua, Morelos | Most probably, a laboratory clone |
| KT963308 | USA_ Dmagna_ABS | Aquatic BioSystems Inc. | Apparently a laboratory culture |
| KT963309 | USA_ Dmagna_NCSU | Aquatic BioSystems Inc. | Apparently a laboratory culture |
| EU702132 | Ontario | Dorset Environmental Science Center, Ontario | Most probably anthropogenic invasion |
| EU702133 | Ontario | Dorset Environmental Science Center, Ontario | Most probably anthropogenic invasion |
