# Supplementary material for: Phylogeography of Daphnia magna Straus (Crustacea: Cladocera) in Northern Eurasia: Evidence for a deep longitudinal split between mitochondrial lineages
Source: PLoS One. 2018 Mar 15;13(3):e0194045. doi: 10.1371/journal.pone.0194045 (PMC5854346; doi:10.1371/journal.pone.0194045)
Supplement: S1 Text — (DOCX) [file pone.0194045.s011.docx]

**S1 Text. Results on one additional mitochondrial and three nuclear gene fragments.**

The analyses were performed separately for each gene using Bayesian phylogenetic analyses (see main text). The number of individuals analyzed was low (between 5 and 19, depending on the fragment), but included individuals with COI-haplotype of both super-clades A and B in all cases (Tables S1, S3). The following number of sequences was analysed: 16S – 14 (14 unique haplotypes); 18S –16 (16); HSP90 – 19 (13); Н3 – 5 (5). The best substitution models found for these genes were: *16S* – HKY+F+G4 (gamma shape alpha: 0.184); 18S – JC+I (proportion of invariable sites: 0.991); *HSP90* – TIM+F+I (proportion of invariable sites: 0.948); *H3* – K2P+I (proportion of invariable sites: 0.859). Unfortunately, polymorphism was relatively low, and the only well-supported separation in the phylogenetic tree was the one between *D. magna* and the outgroup species (S3 Fig and S4 Fig). Even the differentiation between super-clades A and B was not revealed in these threes.
